# Structural and Molecular Modeling Features of P2X Receptors

**DOI:** 10.3390/ijms15034531

**Published:** 2014-03-14

**Authors:** Luiz Anastacio Alves, João Herminio Martins da Silva, Dinarte Neto Moreira Ferreira, Antonio Augusto Fidalgo-Neto, Pedro Celso Nogueira Teixeira, Cristina Alves Magalhães de Souza, Ernesto Raúl Caffarena, Mônica Santos de Freitas

**Affiliations:** 1 Cell Communication Laboratory, Oswaldo Cruz Institute, Oswaldo Cruz Foundation (FIOCRUZ), 4365 Brazil ave, Rio de Janeiro 21045-900, Brazil; E-Mails: dinarteneto@yahoo.com.br (D.N.M.F.); fidalgo@kftox.com (A.A.F.-N.); teixeirapcn@yahoo.com (P.C.N.T.); souzacam@ioc.fiocruz.br (C.A.M.S.); 2 Oswaldo Cruz Foundation (FIOCRUZ) Ceará Avenida Santos Dumont, 5753, Torre Saúde, Sala 1303, Papicu, Fortaleza-CE, CEP 60180-900, Brazil; E-Mail: jhms@fiocruz.br; 3 Scientific Computation Program, Oswaldo Cruz Foundation (FIOCRUZ), 4365 Brazil ave, Rio de Janeiro 21045-900, Brazil; E-Mail: ernesto@fiocruz.br; 4 National Nuclear Magnetic Resonance Center (CNRMN), The National Institute of Science and Technology for Structural Biology and Bioimaging, Institute of Medical Biochemistry Leopoldo de Meis, Federal University of Rio de Janeiro (UFRJ), Carlos Chagas Filho, 373, Rio de Janeiro 21941-901, Brazil; E-Mail: msfreitas@bioqmed.ufrj.br

**Keywords:** P2X_7_ receptor, ion channel activity, patch-clamp

## Abstract

Currently, adenosine 5′-triphosphate (ATP) is recognized as the extracellular messenger that acts through P2 receptors. P2 receptors are divided into two subtypes: P2Y metabotropic receptors and P2X ionotropic receptors, both of which are found in virtually all mammalian cell types studied. Due to the difficulty in studying membrane protein structures by X-ray crystallography or NMR techniques, there is little information about these structures available in the literature. Two structures of the P2X4 receptor in truncated form have been solved by crystallography. Molecular modeling has proven to be an excellent tool for studying ionotropic receptors. Recently, modeling studies carried out on P2X receptors have advanced our knowledge of the P2X receptor structure-function relationships. This review presents a brief history of ion channel structural studies and shows how modeling approaches can be used to address relevant questions about P2X receptors.

## 1. Introduction

The first indication that adenosine 5′-triphosphate (ATP) could be a signaling molecule (neurotransmitter) was provided by Holton and Holton in 1954 in a study of sensory neurons [1]. The identification of ATP as the neurotransmitter in non-adrenergic, non-cholinergic inhibitory nerves in the guinea pig was crucial for the concept of ATP as a signaling molecule [2]. ATP receptors have since been identified in almost all cells and are classified as P2 purinergic receptors, divided into two groups: Ionotropic (P2X) and metabotropic receptors (P2Y). Three subclasses of purine and pyrimidine receptors have been identified: P1 adenosine receptors (A1, A2a, A2b and A3), P2X ionotropic nucleotide receptors (seven subtypes) and P2Y metabotropic receptors (eight subtypes). The P2X receptor subfamily is composed of ATP-gated ion channels and includes seven members (P2X1-7) [3]. This review will focus on structure and modeling of P2X receptors.

## 2. General Principles of Ion Channels

Ion channels are integral membrane proteins that are vital for living organisms. They provide a low-energy barrier for ions and certain hydrophilic molecules to pass through hydrophobic cellular membranes with transport rates close to the diffusion limit, and participate in cell-cell communication and signaling, osmotic stress responses and muscle contraction. Ion channels also play a central role in the primary senses of animals, such as taste, hearing, and touch. Approximately 40 years ago, the existence of ion channels was controversial: They were not regarded as molecular entities, despite the evidence for pores from measurements of the electrical properties of the cell membrane. The concept of ion channels was developed to explain how biological membranes exhibit electrical conductance to selected ions and how this permeability is regulated and changed locally and rapidly by electrical, chemical or mechanical stimuli [4].

In 1952, Hodgkin and Huxley published their quantitative description of action potential propagation in the giant axon of squid [5]. Although they described accurately the changes in membrane Na^+^ and K^+^ permeability that drive changes in membrane potential, they could not determine how ions crossed the membrane and suggested that a molecular entity might mediate this phenomenon. Afterwards, improvements in biochemical techniques allowed scientists to isolate membrane fragments from the electric organ of the fish *Torpedo marmorata*; in this organ, the nicotinic acetylcholine receptor represents a remarkable 30% to 40% of the total protein content [6]. These preparations allowed many groups to study the biochemical and biophysical properties of this new molecular entity, the ligand-gated ion channel (LGIC), and has enabled the first X-ray diffraction studies of an LGIC [7].

In the 1970s, a structure with a filter was proposed to explain the selectivity of ion channels. Ion channel structure determination by X-ray crystallography became feasible after technological advances in sample preparation, such as isolation and purification of membrane proteins. In addition, single-channel patch-clamp measurements demonstrated that the flow of ions was mediated by pores, and that the membrane conductance of an ion channel depended on multiple binding sites or receptors. These observations established that ion channels were in fact molecular entities.

The elucidation of the structure of the prokaryotic potassium ion channel (KcsAa) from the soil bacterium *Streptomyces lividans* at a resolution of 2.0 Å was an important achievement to further understand ion channel structure. The selectivity filter is a narrow, 12 Å-long segment of the pore that is lined with carbonyl oxygen atoms [8]. These atoms act as surrogate water molecules, thereby allowing potassium ions to shed their hydration shell and enter the pore. In this model, two potassium ions bind with selectivity to the filter at once, most likely with a single intervening water molecule. Electrostatic repulsion between the ions inside the filter prevents the potassium ions from binding too tightly, allowing them to conduct at a high rate [9]. Recently, Jensen *et al*. proposed such a model for voltage gating that included both the activated-to-deactivated state transition as well as some key steps of the resting-to-activated-state transition [10]. They applied all-atom molecular dynamics simulations to provide insight into how a voltage-gated potassium channel switches between activated and deactivated states.

## 3. Molecular Modeling as an Essential Tool for Investigating Ion Channels

The history of the study of ion channels is linked to that of mathematical modeling in biology; Hodgkin and Huxley solved complex differential equations for the action potential. Since then, computational techniques have evolved greatly, allowing the description of several phenomena that would be impossible to measure or detect with conventional laboratory manipulations.

At the most fundamental level, ion selectivity must result from an exquisite balance of interactions. Molecular dynamics and free-energy analyses are important tools for investigating the ion selectivity of the channels. Moreover, the narrow pore is perfectly suited to providing a cavity of the appropriate size to fit the selected ion [8]. Similarly, the simplest explanation of ion selectivity relies primarily on the protein structure and proposes that the protein-binding site provides a cavity of the appropriate size for one ion and is unable (for structural reasons) to adapt to an ion of a different size [10].

Homology modeling has been used to study the physiology and structure-function relationships of ion channels. The conformational dynamics of the nicotinic acetylcholine receptor (nAChR) were explored by immersing a pore-lining region of nAChR in a mimetic membrane and conducting 15-ns molecular dynamics simulations [11,12]. The same group also studied the changes in the M2 helices of nAChR in different environments, this segment represents the second transmembrane domain of this receptor. When immersed in water, the M2 helix partially unfolds, forming a hinge-like structure close to a critical Leu residue that has been associated with the mechanism of ion-channel gating [12]. Molecular dynamics simulations were also used to analyze the conformational dynamics of the S6 helix hinge in models of a portion of a Kv channel that is associated with channel gating. These simulations suggested a channel-gating model that involved the S6 fold proximal to the PVP (proline-valine-proline) motif, a motif that is conserved among potassium channels. The simulations led to a model with a three-hinged mechanism for gating. The same study also proposed that gate opening is dependent on the expansion or an increase in the polarity of the side chain situated in the narrowest region of the gate.

### 3.1. Ligand-Gated Ion Channels

**Figure 1.**
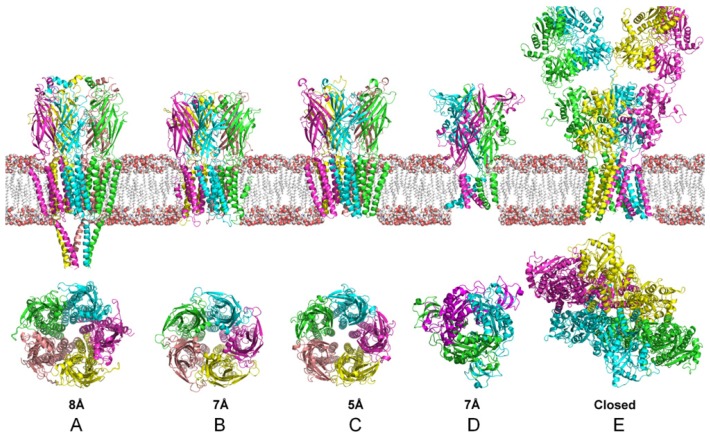
Cartoon representation of the three different families of ion channels. Pentameric: (**A**) Structure of the nicotinic acetylcholine receptor (PDB code: 2BG9); (**B**) A ligand-gated ion channel from *Erwinia chrysanthemi* (PDB code 2VL0); (**C**) An open pore conformation of a bacterial ligand-gated ion channel(PDB code 3EAM); Trimeric: (**D**) Crystal structure of the adenosine adenosine 5′-triphosphate(ATP)-gated zebrafish P2X4 ion channel in agonist-bound (PDB code 4DW1); Tetrameric: (**E**) Structure of the AMPA subtype ionotropic glutamate receptor (PDB code 3KG2). The figure also includes a representation of the biological membrane showing the actual proportions of the transmembrane channel. The images below the membrane represent the top view, taking the membrane as a reference. The numbers show the approximate diameter of the narrowest region of the pore.

There are many ways to classify LGICs. For example the International Union of Basic and Clinical Pharmacology (IUPHAR) recommends classifying them by their ligands. However, in this review we will classify them by their quaternary structure (Figure 1). The first two classes are the pentameric [13] and the tetrameric superfamily [14]. The third class contains trimeric ion channels, which include P2X receptors and the acid-sensing ion channels [15]. These classes are summarized in Table 1.

### 3.2. Pentameric Receptors

Pentameric receptors are the best-characterized receptors; they are also called Cys-loop receptors [13]. These receptors comprise (1) excitatory and cation-selective, as represented by nAChR, 5-hydroxytryptamine type 3 receptor (5-HT3R) and Zn^2+^-activated ion channel receptor (ZACR); and (2) inhibitory anion-selective receptors, as represented by γ-aminobutyric acid receptors (GABA_A_Rs), glycine receptor (GyR) and its invertebrate equivalent, the glutamate-gated chloride channel receptor (GluCir) [16–18].

Pentameric receptors are composed of five identical subunits. These receptors possess a characteristic loop formed by two cysteine (Cys) residues connected by an intrasubunit disulfide bridge. These residues also comprise an extracellular domain (*N*- and *C*-terminal) that contains specific recognition sites for transmitters and additional sites for allosteric regulators such as benzodiazepines, alcohols and anesthetics [18]. The M2 domain lines the channel, formed by association of the five M2 segments. nAChr was the first LGIC identified at neuromuscular junctions [19]. Electrophysiological recordings after exposure to the acetylcholine (ACh) agonist showed a rapid inward current (activation) followed by a progressive decline upon sustained agonist application (desensitization). These results demonstrated that nAChRs have three states: Closed at rest, open during activation and closed by desensitization. However, within the same family, different receptor subtypes can display different properties. In the nAChR family, the heteromeric receptors at neuromuscular junctions display sustained currents in response to ACh, whereas the homomeric α7 nAChR exhibits a rapid desensitization [19].

### 3.3. Tetrameric Receptors

The members of the tetrameric receptor superfamily are excitatory and cation-selective [14]. The GluR gene family comprises tetrameric ionotropic receptors defined by their specificity for (*N*-methyl-d-aspartate (NMDA), α-amino-3-hydroxy-5-methyl-4-isoxazolepropionic acid (AMPA) and kainate. Glutamate is the chemical transmitter of excitatory synapses in the central nervous system. iGluRs are fundamental to neurotransmission at excitatory synapses and are implicated in nearly all aspects of nervous system development and function [20]. Each subunit of the tetrameric complex has an extracellular domain that contains specific recognition sites for ligandbinding, an extracellular *N*-terminal domain for putative regulatory interactions, an intracellular *C*-terminal domain and transmembrane domains that constitute the ion channel [21]. In contrast to the Cys-loop receptors, in which the ligand-binding sites are formed at the interface between subunits, the ligand-binding sites in iGluRs are located in the core of each subunit.

AMPARs, NMDARs and kainate receptors are related in amino-acid sequence but diverge with respect to their functions [20]. NMDARs do not exhibit the kinetics of activation, deactivation and desensitization found in obligate heterotetrameric arrangements. NMDARs are slower, corresponding to a molecular process occurring on a timescale of tens to hundreds of milliseconds. Whereas AMPARs and kainate receptors only need glutamate for activation, NMDARs also require membrane depolarization to relieve the magnesium block [22], as well as glycine binding [23].

### 3.4. Trimeric Receptors

The last family is the excitatory ATP-gated P2X receptors (P2XRs). Seven genes (P2XR1-7) encoding P2XR subunits (P2X1-7) are known in vertebrate species [3]. However, there is no evidence of P2XRs in prokaryotes. The majority of the subtypes are nonselective cation channels with high Ca^2+^ permeability. An exception is P2X5R, which is permeable to anions such as Cl^−^. These receptors play important roles in cell-cell communication by mediating neuromodulation with ATP rapidly gating channels and triggering transmembrane fluxes of selective ions as well as in immune responses.

P2XR channels are composed of three subunits, which assemble as either homomeric or heteromeric complexes. Each subunit has two transmembrane domains, with a large Cys-rich extracellular domain (~280 residues) and intracellular *C*- and *N*-termini. P2XRs adopt relatively simple architectures for LGICs, in contrast to eukaryotic GluRs, which form tetramers, and the pentameric receptors (Figure 1) [24,25]. The trimeric structure of P2XRs more closely resembles that of epithelial Na channels (ENaC), the acid-sensing ion channels (ASICs) and the mechanosensitive channels (Mscs). The second transmembrane domain (TM2) appears to play a major role in P2XR channel formation, but TM1 also participates in the integral function of these receptors [26–29].

As described for the pentameric channels, each homomeric P2XR also displays distinct functional properties. In general, the gating of P2XRs consists of three phases: a rapid phase of inward current induced by agonist application (activation phase), a slow decay phase in the presence of the agonist (desensitization phase) and a rapid decay of current after ATP removal (deactivation phase). The P2XRs differ primarily in their sensitivity for agonists and their desensitization rates [30,31].

Most P2XRs, once activated by their native agonist after exposure for a range of millisecond durations, open a small cationic channel, although some channels (P2X2R, P2X4R and P2X7R) also allow the passage of larger molecules, such as Lucifer Yellow, YOPRO and propidium iodide [32,33].

Recently, Gonzales *et al.* drew a parallel between the architecture and the mechanism of the ATP-gated P2X (1-7subtypes) receptors and the acid-sensing ion channels [15]. The authors described that P2X1 and P2X3 subtypes of P2X family and the ASIC1a show rapid and profound desensitization, whereas P2X2, P2X4, P2X7 and ASIC2a yield slower, more incomplete desensitization. According to this information, the ASICs may be classified in the future as LGICs in The International Union of Basic and Clinical Pharmacology (IUPHAR). At present, ASICs are classified as members of the sodium channels family, despite being gated by protons.

## 4. Structural and Modeling Aspects of P2XRs

In this session, we summarize some P2X receptors structure-function relationships obtained in most of cases by site-directed mutagenesis and homology models.

### 4.1. P2X1 Receptor

The P2X1 receptor binding site environment seems to be formed by aromatic residues, like other ATP-binding proteins. This environment may confer selectivity for the nucleotide base and ribose ring. As expected, there are also positively charged residues [34], which interact with the negatively charged phosphate groups. Insights into the residues that contribute to ATP binding have been provided by site-directed mutagenesis. A recent sulfonate accessibility study demonstrated that residues in the putative ATP binding site are not accessible to methanosulfonates when ATP is bound to the receptor, as in zebrafish P2X4 receptor (zfP2X4), in which conformational changes that occur when ATP binding leads to the opening of the receptor hide the sulfonate binding site (ATP itself might also block the binding site). A homology model of the P2X1 receptor was constructed and utilized in combination with electron microscopy observations. This approach demonstrated that conformational changes that occur when the receptor enters the open state lead to the exposure of putative fenestrations next to the lower vestibule through which the ions can pass; this pattern is also expected in zfP2X4 [35,36].

One outstanding feature of P2X1 is that fast desensitization during ATP application, which appears to be due to the interaction between the hydrophobic transmembrane domains [30]. Werner *et al.* created chimeric receptors that replaced the transmembrane domains of the rapidly desensitized P2X1 receptor with those of the slowly desensitized P2X2 receptor [37]. Electrophysiological analysis revealed that the replacement of the P2X1 transmembrane domains with the P2X2 transmembrane domains resulted in slow desensitization. Furthermore, when the chimera had one transmembrane domain from each of the receptors, an intermediate desensitization pattern emerged, clear evidence that transmembrane domains interactions are directly involved in the desensitization mechanism.

The P2X1 receptor can assemble as heterotrimers with P2X4 subunits when heterologously expressed in *X. laevis*. This assembly, which occurs under physiological conditions since P2X1 and P2X4 are co-localized in the plasma membrane, might explain the different patterns of P2X1 currents recorded in some tissues in comparison with homomeric P2X1 currents expressed in a heterologous system. For example, Nicke *et al.* demonstrated that the co-injection of P2X1 and P2X4 mRNA in *X. laevis* leads to the formation of a channel with an EC50 similar to that observed for homomeric P2X1 receptors but a desensitization curve similar to that for P2X4 [38]. In addition, this current could be blocked by suramin, which has no effect on the homomeric P2X1 receptor (but can block the P2X4 receptor).

Allsopp *et al.* used a homology model of P2X1 receptor to explain the results of mutagenesis studies of the putative ATP binding site [39]. Some substituted residues were able to reduce ATP potency directly by affecting ATP binding or disturbing the channel gating kinetics. Using a radiolabeled ATP binding assay, Allsopp *et al*. determined that residues K68, K70 and F92 are likely directly involved in ATP binding. The authors then tested this hypothesis through docking experiments with a homology model. The best-scored ATP binding patterns supported the importance of the positively charged residues K68, K70, R292and K309 for the interaction with the negatively charged oxygens of the phosphate groups of ATP (there was a contribution from N290, but it was due to the amine group, not the side chain). The model also revealed that the adenine ring interacts with a hydrophobic backbone comprising Val67, Phe100, Phe289, Phe291 and Phe92 and that the ribose ring interacts with Phe92 (mutations of which also decrease ATP potency) and the carboxyl group of Ala88.

Roberts *et al.* also used a combination of mutagenesis studies and a homology model to gain insight into the gating kinetics of the P2X1 receptor [40]. Following the observations of Allsopp *et al.*, Roberts *et al.* demonstrated that the putative ATP binding site is localized 40 Å from the transmembrane domains and that conformational changes occur throughout the receptor, resulting in domain rearrangement. There are β-strands between the putative ATP binding site and the transmembrane domains in the P2X1 receptor homology model. Cysteine biotinylation accessibility experiments demonstrated that these β-strands are inaccessible even when the receptor is bound to ATP, but when the authors artificially connected β-strands from different subunits assumed to be close in the trimeric architecture of the receptor by cysteine substitution cross-linking, the receptor became non-functional, suggesting that movement in this interface underlies the P2X1 gating process. All data are in agreement with the homology model used to explain these results. These studies clearly demonstrate the relevance of homology models to interpret mutagenesis studies in the absence of a crystal structure. After these homology studies were published, the crystal structure of zfP2X4R in the agonist-bound state was determined [35], revealing a pattern of conformational changes highly similar to that predicted by modeling.

### 4.2. P2X2 Receptor

The homomeric P2X2 receptor is expressed in primary sensory neurons, which are responsive to injuries. The P2X2 receptor is expressed on medium- and large-diameter sensory neurons and exhibits slow desensitization rates [30,41,42]. A proposed model for a single channel formed by homomeric P2X2 indicates three ATP molecules binding in a cooperative fashion, in which two open states are connected to a closed state [41]. The ATP binding site has been proposed to be composed of K69, K71, N140, L186, K188, N288, F289, R290 and K308. The lysines belonging to the ATP binding site seem to be well conserved among P2X receptors, suggesting an important role in channel activity. K69 binds simultaneously to the α, β and γ phosphate groups, and its absence or slight perturbation can abolish receptor functionality [43]. Although no antagonist selective for the P2X2 receptor has been identified, some divalent cations play a role in decreasing the affinity to ATP. The cation-amino acid interaction might occur via an electrostatic attraction, making the binding site less accessible to the agonist. In fact, it has been proposed that the antagonist should be larger than the agonist to bind in an open-jaw fashion as in the Apo closed-channel state. In contrast, the ATP-receptor binding site should be kept in a closed-jaw state, releasing conformational changes that result in the open-channel state [44,45].

A structural model for P2X2 was obtained in 2013 by comparing the crystallographic structure for the Apo and ATP-bound truncated zebrafish P2X4 receptor. The models illustrated only slight changes in the upper body of the receptor but there was a major molecular movement with a deviation of approximately 8 Å between the Apo and ATP-bound structures [44]. The transmembrane domains seem to play a role in the permeability changes in P2X2 because the interface between the transmembrane domains is linked to conformational rearrangements between the pore channel states. In 1999, Torres and co-workers obtained evidence that the transmembrane regions participate in the homo/hetero-oligomeric association that is believed to be important for pore formation [46]. The TM2 segment has been identified as responsible for ion permeation and trimerization because the region spanning residues 304 to 362 in this segment is critical for that association.

Recently, Rothwell *et al.* showed that MTS (propyl-methanothiosulfonate) can evoke ATP independent currents in P2X2 receptor when cysteine substitution was made in the putative outer edge of P2X2 receptor TM domains. This study suggests that close package between TM helices and lipids in the membrane in P2X2 closed state conformation was disrupted by covalent binding of MTS to mutated cysteine residues. Furthermore, MTS and other lipophilic methanothisulfonated compounds, but not polar ones, have similar effects, corroborating that the MTS effect occurs in the membrane environment. Using a homology model of P2X2 receptor based on zfP2X4 receptor the author could identify the putative positions of residues of the edge of the TM domains [47].

### 4.3. P2X3 Receptor

P2X3 is primarily expressed on small- and medium-diameter sensory neurons [30,48], undergoes rapid desensitization and responds to differing extents to the presence of modified ATP agonists [49,50]. The ATP binding site in the human P2X3 receptor is thought to comprise the residues K63, G66, T172, K176, N177, S275, N279, R281, R295 and K299 [51]. It has been proposed that conformational changes triggered by agonist-receptor binding affect the pore dynamics between the closed and open states. However, other proposals support the presence of structural fluctuations that are limited by the energy barriers between the states. The agonist plays a role in stabilizing one of these structures. In fact, even in the absence of the agonist molecule, it is possible to observe a pore population displaying the open state. In the P2X3 receptor, the residue D266 has been linked to allosteric modulation in which the extracellular calcium ion acts as an allosteric effector. In 2011, it was proposed that residue D266 plays a stabilizing role in the region of the left flipper, which has been implicated in agonist binding [51]. Because allosteric modulation is important for either increasing or decreasing receptor activity, allosteric effectors should be evaluated not only locally but also globally, from a more complex perspective in which more than one allosteric effector may be interacting with the same molecular target. From this perspective, cross-effects should be evaluated in an effort to identify favorable synergic effects that may be applicable as a therapeutic strategy.

### 4.4. P2X2/3 Receptors

Like many of the P2X receptors, the P2X2 and P2X3 receptors are found in either homomeric or heteromeric configurations. Hetero-oligomeric interactions do not occur promiscuously, as in the case of P2X receptors, but in a selective manner that may be dependent on the cluster of helices formed by six transmembrane segments, which are less rigid than in P2XRs but have lower affinity, as the pore should dilate by 3 Å, as previously suggested [51]. As examples of the heteromeric configurations of P2X receptors, P2X1 can combine with P2X2, P2X5 or P2X6, and P2X2 can form associations with either P2X3, P2X4 or P2X5. The heteromeric P2X2/3 receptor exhibits pharmacological behavior similar to that of P2X3 but a desensitization profile similar to that of P2X2R [52]. Although P2X3 and P2X2/3 are the most abundant P2X receptors in the dorsal root ganglia neurons, the response to ATP in nodose sensory neurons is mediated by P2X2 and P2X2/3 [44,49]. The differences between P2X2 and P2X3 could be better understood if they were linked to a structural profile. However, the lack of structural information concerning these receptors hampers extensive correlations between structural elements and receptor activity. Some insights have been obtained from comparisons of P2X and the already solved P2X4 receptor structure. P2X2, P2X3 and P2X2/3 contain a huge ectodomain, two transmembrane regions and intracellular *N*- and *C*-termini. The electrostatic surface might dictate the affinity of the agonist for the binding site, which is dependent on the amino-acid sequence. Despite the high amino-acid conservation between P2X2 and P2X3, local differences may have an impact on whole-receptor activity. In pore formation, the ion selectivity of the P2X channel is activation-dependent, which suggests a necessity for applying dynamic filters and changes in conformation among preferential ions.

### 4.5. P2X4 Receptor

**Figure 2.**
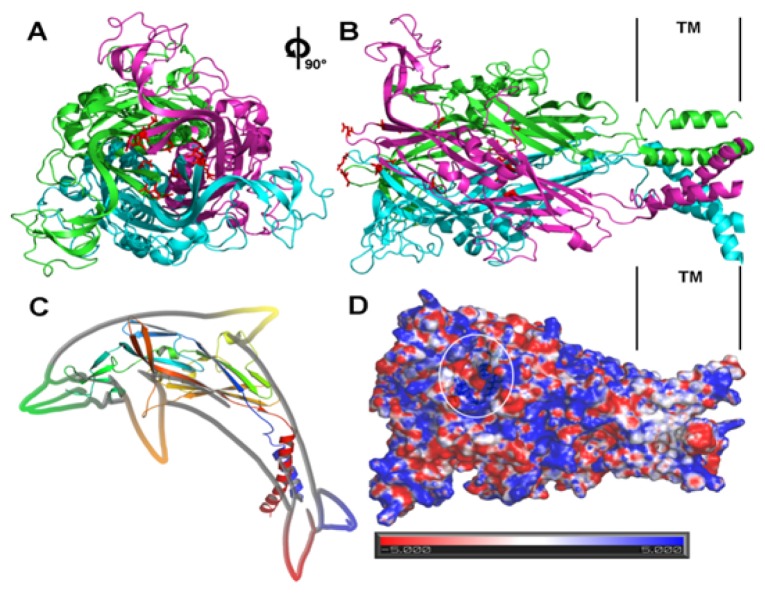
Top (**A**) and side (**B**) view representation of a P2X4 ion channel, taking the membrane as reference. Residues D91, D99, D307, E310 and D323 lie along the assumed ion pathway and are shown in red. Three monomeric units (pink, green and blue form a trimeric configuration in which these negatively charged amino acids can form a strong positive gradient that supports ion migration into the cell; (**C**) Dolphin like shape of P2X4 subunit; The highlighted region in (**D**) shows the putative ATP binding site (N296, R298, K316, K70, K72, F188 and T189), as suggested by Hattori *et al.* [21]. The binding cleft is located between two adjacent chains. Figure created using APBS (APBS version 1.3 [52]) and PyMOL (The PyMOL Molecular Graphics System version 1.5.0.4 [53]).

The P2X4 receptor is one of the most studied P2X subtypes, and the elucidation of its structure was considered a major advance in the understanding of the molecular mechanisms of the 3D structure and channel gating of P2X receptors [35,36]. Kawate *et al.* demonstrated in a brilliant manner the 3D structure of the incomplete P2X4 receptor in the Apo state through X-ray crystallographic techniques (resolution 3.1 Å) [36]. This work confirmed the trimeric structure and subunit topology (two transmembrane segments bound to a large extracellular loop, consisting mainly of beta-sheet motifs) and an hourglass-shaped transmembrane domain consisting of six alpha-helical segments (three TM1 and three TM2), with the three TM2 helices crossing half of its length in the center of the pore and sloping 45° with respect to the membrane axis, thus constraining the channel gate. Hattori *et al.* expanded our knowledge of P2X receptor activation by identifying three putative ATP binding sites located between the head and the left dorsal fin of the adjacent subunit (referring to the “dolphin-like” structure of P2X4 described by Kawate-illustrated in Figure 2) and a lateral fenestration to the channel gate next to the extracellular vestibule [35].

The 3D structure of zebrafish P2X4 (zfP2X4) enabled the construction of models (in the Apo state) of the other P2X subtypes through homology modeling. Li *et al.* studied the transitions between the closed and open states of the transmembrane domain, allowing for a realistic spatial overview of cysteine accessibility studies using Ag^+^ and Cd^+^ as blocking elements [29]. These experiments revealed a general rearrangement (not punctual) of the transmembrane domains upon the transition from the closed to the open state. In general, the results of these studies can be summarized in a model in which a wide enlargement of the intracellular vestibule occurs following a narrowing of the extracellular vestibulum. Thus, the receptor changes from an hourglass-like shape to a funnel-like shape based on the reorientation of the TM2 longitudinal axis to the membrane axis, followed by a slight rotation around its own axis, as revealed in the ATP-bound zfP2X4 structure [35].

It is important to note that both 3D structures (closed or agonist-bound) are truncated (but functional) and lack the *N*- and *C*-termini, with resolutions of 2.8 and 2.9 Å, respectively. The availability of the receptor structure in the open state permitted the identification of the residues that contribute to the ATP binding site and the stereochemistry of this binding, confirming the predictions made in previous studies. The comparison between the closed and open states led to the formulation of an elegant kinetic model that revealed how conformational alterations triggered by ATP promote pore dilation in the plasma membrane [35]. The “head region” of the dolphin-like subunit appears to be rigid because the differences in this region between the closed and open states are small. By contrast, the “lower body” region undergoes more drastic conformational changes that result in the rearrangement of the transmembrane helices, leading to the opening of the ion-conducting pathway in the transmembrane domains.

In simple terms, pore dilation results in a slight bend in the dolphin lower body triggered by ATP when it closes the ATP binding site. This bend has a hinge-like effect on the TM1 and TM2 segments, shifting them sharply outward from the pore center. This movement leads to the expansion of the extracellular vestibulum like a camera diaphragm, opening the pore. Another interesting result reported by Hattori was the wide enlargement of the upper vestibulum, which indicated that the lateral fenestration should be the ion pathway to the pore in the opening process. However, it is important to highlight that the work of Hattori is based on crystallographic studies of an incomplete zfP2X4 in which the *N*- and *C*-termini are missing, which may affect the receptor dynamics. It has been shown that the P2X7 *C*-terminus is a prerequisite for opening the large-conductance pore. Hattori determined that the modified version of zfP2X4 could not form the large-conductance pore [35].

### 4.6. P2X5 Receptor

Among P2X receptors, the P2X5 structure has been barely studied. This receptor is the least conserved with respect to amino-acid sequence in the P2X family and is the sole anion-selective subtype [31,54]. However, studies have shown that P2X5 receptors have features similar to those of the other P2X subtypes, such as the relevance of the second transmembrane domain to the ion pathway across the membrane and its trimeric structure. In humans, the P2X5 receptor is predominantly expressed as a nonfunctional isoform that is restricted to the cytosol. This expression pattern is unique to humans and is not found in other mammals, in which only the functional isoform is expressed. The relevance of its nonfunctional expression in humans in an evolutionary context remains unknown.

The second transmembrane segment is important for receptor oligomerization and, as expected, for the ion-conduction pathway. Nevertheless, only a small segment in the second transmembrane domain seems to be important for the passage of ions, because only mutations in a small portion of these domains are able to block the ATP-gate current [54].

The P2X5 receptor can form heterotrimers with P2X1 [55] and P2X2 [56]. The recently described P2X2/P2X5 heterotrimer can take up YO-PRO and promotes membrane blebbing like the P2X7 receptor, representing a novel pattern in the receptor family. Moreover, this heterotrimer can be found *in vivo*, thus representing a new way to sense ATP in the nervous system.

There have been no studies of the ATP binding site or channel gating in P2X5; thus, we cannot draw additional conclusions about structure-function relationships in the P2X5 receptor.

### 4.7. P2X6 Receptor

Very little is known about the 3D structure of the P2X6 receptor, primarily because this receptor does not homo-oligomerize, which prevents the formation of the channel in a homomeric form; this receptor is only functional when oligomerized with P2X2 [57] or P2X4 [58]. Site-directed mutagenesis studies by Ormond *et al.* revealed that a region of the uncharged *N*-terminal is crucial for its oligomerization and export from the endoplasmic reticulum [59]. When this region was removed or its charge was altered, the P2X6 homotrimer was not formed, and the protein was subjected to glycosylation and exported to the cytoplasmic membrane, even though it was not functional.

### 4.8. P2X7 Receptor

The overall structure of P2X7 is similar to that of the other P2X family members except for the *C*-terminus [60]. The long *C*-terminal sequence (239 aa) of P2X7 is unique among P2X family members and encompasses several other protein and lipid recognition motifs as well as a cysteine-rich domain. This domain also appears to be important for interactions with lipopolysaccharides (LPS) because it includes a conserved LPS-binding domain. *In vitro* experiments revealed that the ability of LPS to activate extracellular kinases is blocked by the presence of peptides derived from this P2X7 domain. These data suggest that the P2X7 receptor *C*-terminus is able to coordinate events related to signal transduction during LPS activity [61]. In addition, the mouse P2X7 receptor can be activated by NAD through ADP-ribosyltransferase in a process that involves the ADP-ribosylation of Arg125 and culminates with channel activation [62]. The residues Cys572, Arg574 and Phe581, which are located in the *C*-terminus, prevent surface expression when mutated to Gly [63]. The atomic coordinates of P2X7 are unknown, but extensive structure-activity relationship (SAR) studies have led to the discovery of several P2X7 antagonists, which were widely reviewed recently [24,64–66]. Although the structure of the zfP2X4 receptor has been elucidated, it does not provide a clear explanation of the differences in ligand potency between P2X superfamily receptors. In addition, P2X4 is much more sensitive to ATP than P2X7.

Homology models of P2X7 recently shed light on the details of P2X7 ATP binding and ligand recognition [66]. The putative ATP binding site is located between two adjacent subunits and most of its residues are positively charged (Lys64, Lys66 and Lys197 in one subunit and Arg294 and Lys311 in the other). Mutagenesis studies have suggested that the mutation L191P in the human P2X7R impairs receptor function [67]. In addition, residues around the ATP binding site appear to contribute to the functional properties of human P2X7R. The K145 residue is highly conserved among mammalian P2X7 proteins and is located in the head domain and close to the ATP binding site, which may favor the access of agonists to the binding site. A charge-neutralizing K145A mutation in rat P2X7R decreased BzATP sensitivity [68]. Conversely, the R276A mutation, which is also located in the vicinity of the ATP binding site, enhanced sensitivity to ATP and BzATP [69]. This residue, along with R277, appears to be involved in conformational changes following ion-channel gating or receptor activation/deactivation [27,48].

**Figure 3.**
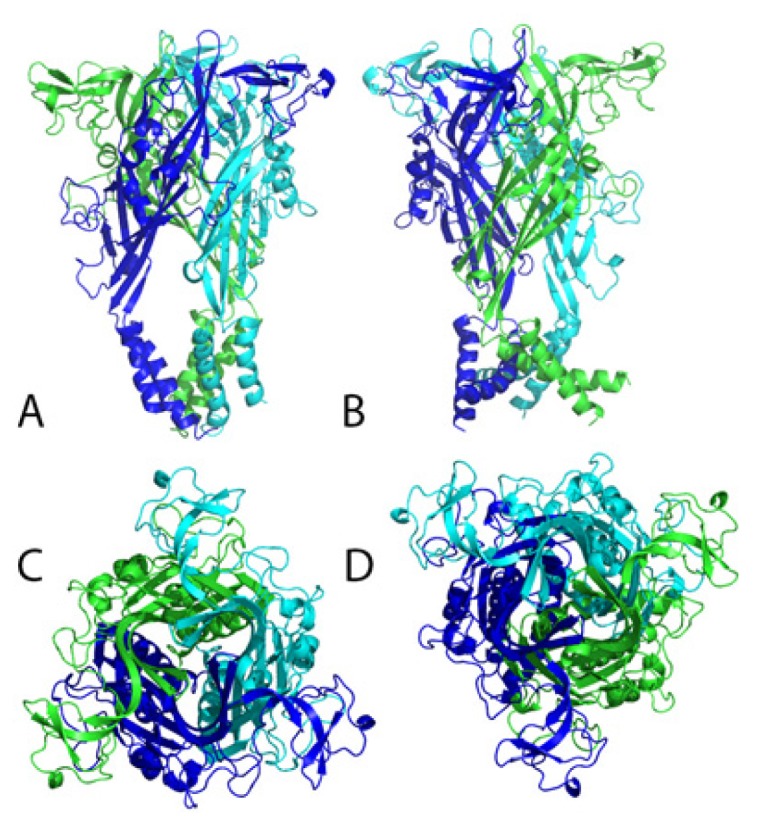
(**A**) and (**B**) are shown the Open (ATP bound) and closed side view of P2X7R built from the P2XR4 structure, respectively; (**C**) and (**D**) are shown the Open and closed top view (from outside the cell membrane), showing the diameter of the gate, respectively.

**Figure 4.**
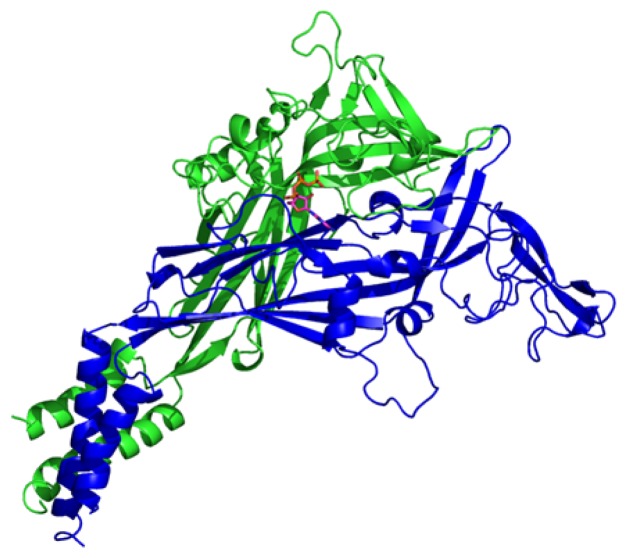
Side view of two adjacent units, showing a putative ATP binding site, transposed from the P2X4 crystal structure.

The sequence alignment between human P2X7R (UniprotKB code Q99572) and the P2X4 (PDB code 4DW1) receptors was done and afterwards a comparative 3D structural model was built. The identity between the 2 primary sequences was 47.3%, while the similarity was 64.8% (data not shown). The 4DW1 crystal was found to be a good template for P2X7R (Figure 3). The ATP binding site was transposed to the P2X7 structure, keeping the structural alignment between target and template. After the stereochemical validation, 95.6% of the residues were found to be in the most favorable regions, while, for the template used (4DW1), 96% were found in the same region. The Z-score reached 7.55, compatible with similar structures obtained through X-ray scattering. The energies of the amino acids, as a function of its position, calculated with Prosa, was lower than 0 to all the residues but a group of 5, in the beginning of the *N* terminus. We submitted also the template, obtaining a similar energy profile. The verify3D Z-score was −2.73, calculated by the Proteins Structure Validation Suite (http://psvs-1_5-dev.nesg.org/). Also in verify3D, more than 70% of the residues scored >0.2. The trimeric structure was obtained aligning the P2X7R monomeric units into the 4DW1 biological assembly. The putative ATP surrounding (Figure 4) area is mostly composed by positive and hydrophobic residues (3 Lys, 2 Val, Ile, Leu, Tyr and Thr).

**Figure 5.**
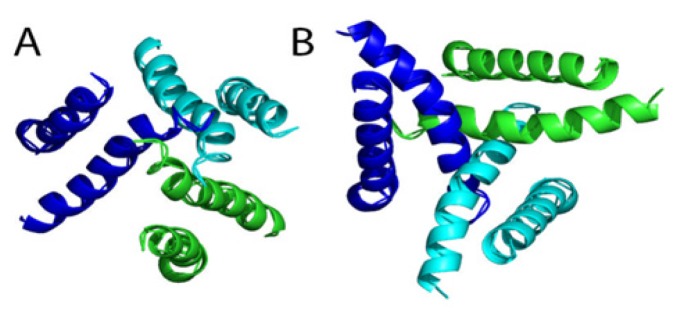
Detailed views of the open (**A**) and closed (**B**) P2X7R channel. The measured diameter of the channel in the open structure is about 7.6 Ǻ, but wider openings may be attained.

The called “tail” of P2X7R shows a possibly similar opening mechanism. While the ATP is undocked, the gate is totally closed, blocking the passage of any Ion or even solvent. The open gate has a diameter of 7.6 Å, which allows the passage of some ions and small cofactors (Figure 5).

## 5. Conclusions

Despite the great advances of the last 60 years since the work of Hodgkin and Huxley, the molecular details and function of ion channels remain incompletely elucidated. Crystallographic techniques, molecular biology and bioinformatics have contributed to the elucidation of this important group of structures. As unanswered questions about ion channels are resolved, important pathophysiological processes are being elucidated, resulting in more effective therapies and promising drugs under development.

Nevertheless, in the P2X area, the mechanism of the ion channel formation and in some cases the pore formation (the high-conductance channel that allows passage of dyes) need to be deciphered in order to pave the way for the discovery of new medicines that act on P2XRs. The molecular modeling and structural studies will be crucial for understanding the transition from a low-conductance state to a high-conductance state that occurs in some members of the P2X family.

## Figures and Tables

**Table 1.** Ligand-gated ion channels (LGICs) and their ligands and permeant ions. (GABA (γ-aminobutyric acid), ACh (acetylcholine), 5-HT (serotonine), AMPA (α-Amino-3- hydroxy-5-methyl-4-isoxazolepropionic acid), NMDA (*N*-Methyl-d-aspartate), ATP (adenosine 5′-triphosphate).

Ligand	Pentameric receptors	Permeant ions
Glycine	GyR (vertebrates)	anions
GABA	GABA_A_R (vertebrates)	anions
ACh	nAChR (vertebrates)	cations
5-HT	5-HT3R (vertebrates)	cations
Zn^2+^	ZACR (vertebrates)	cations
Glutamate	GluR (invertebrates)	cations
5-HT	MOD-1 (invertebrates)	anions
GABA	EXP-1 (invertebrates)	cations
	Tetrameric-Ionotropic glutamate receptors	
Glutamate	iGluRs	
Glutamate	AMPARs	
Glycine and Glutamate	Kainate	cations
	NMDARs	
	Trimeric-Purinergic receptors	
ATP	P2XRs	cations
